# The Sicker Sex

**DOI:** 10.1371/journal.ppat.1000267

**Published:** 2009-01-30

**Authors:** Marlene Zuk

**Affiliations:** Department of Biology, University of California, Riverside, California, United States of America; The Fox Chase Cancer Center, United States of America

Arguments about the weaker sex notwithstanding, there is no contest about the identity of the sicker sex—it is males, almost every time. Everyone knows that old age homes have more widows than widowers, but the disparity extends far beyond the elderly. Fewer women than men died in the 1917–1918 influenza epidemic; the differential mortality was not related to World War I, as originally thought, but was global and widespread among ages. Kruger and Nesse [1] compared men's and women's mortality rates for 11 causes of death in men and women from 20 countries, including accidents and homicide as well as infectious and non-infectious diseases, and found that men virtually always die earlier. They concluded, “Being male is now the single largest demographic risk factor for early mortality in developed countries”. Furthermore, in many free-living mammals, males are more likely than females to harbor parasites or to suffer more intensely from their effects. During the mid-20th century, a virtual cottage industry developed in which investigators experimentally infested laboratory rodents with parasites and documented any resulting sex differences in the prevalence or intensity of the infection that developed [2]. Males usually developed higher parasitemia, with castration removing the sex difference. The persistence of these patterns in the laboratory suggests that the sex difference is not merely due to differences in exposure to parasites, with males and females behaving differently in the field and hence incurring different risks of infection, but to an inherent sex difference in vulnerability.

What causes this disparity between the sexes in longevity and parasite susceptibility? Most research has focused on the proximate mechanisms, such as endocrine or immunological pathways, that are immediately responsible for any one cause. Here, I take a different approach. Sex differences in infection rates or mortality may come about for the same reasons as other differences between males and females, such as morphology: selection acts differently on the sexes because they maximize their fitness in different ways. Below I discuss an evolutionary approach to the question of why males so often die sooner and develop more diseases than females [3],[4]. Some researchers are hopeful that the gap between men's and women's lifespans will close as we develop better medical care and education about health risks, but I will argue instead that the disparity is not going away any time soon.

## Living Well, Dying Young

A subset of evolutionary theory called sexual selection holds that females and males usually inherently differ because of how they put resources and effort into the next generation, which is termed parental investment [5],[6]. Female reproductive success is limited by the number of offspring a female can produce and rear. Because they are the sex that supplies the nutrient-rich egg, and often the sex that cares for the young, females will usually leave the most genes in the next generation by having the highest quality young they can; the upper limit to the quantity is usually rather low. Which male they mate with could be very important, because a mistake in the form of poor genes or no help with the young could mean that they have lost their whole breeding effort for an entire year. Males, on the other hand, can leave the most genes in the next generation by fertilizing as many females as possible. Because each mating requires relatively little investment from him, a male who mates with many females sires many more young than a male mating with only one female.

Variance in male reproductive success is thus expected to be higher, on average, than variance in female reproductive success, which in turn selects for what might be termed a “live hard, die young” overall strategy for males, at least with respect to mating behavior. In elephant seals, for example, males battle ferociously for dominance, and a single male may sire more than 90% of the pups in a colony, leaving the vast majority of males with no offspring, while females will virtually always give birth to a single pup.

With regard to parasite susceptibility, these sex differences in reproductive strategy may provide the ultimate selective force behind increased male vulnerability to infections. If males require, for example, testosterone for aggressive behavior and the development of male secondary sexual characters, selection for winning at the high-stakes game males play may override the cost of any immunosuppressive effects of the hormone. Alternatively, increased stress levels in displaying males could influence susceptibility. Sex differences in infection may thus simply reflect the larger pattern of differential selection on the sexes.

Another way to look at this comes from life history theory, which examines the evolution of such life “decisions” as how many offspring a species is expected to reproduce and how large those offspring should be at birth or hatching. The underlying assumption is that organisms have a finite pool of energy or resources to draw from, and therefore must allocate that energy to different tasks. Because the resources used for one function are unavailable to another, trade-offs between traits such as growth rate and body size, or between the size and number of offspring, are expected. Life history theory explains many of the apparently maladaptive features of life; animals cannot be good at everything. Along these lines, despite the obvious advantage of being resistant to disease, susceptibility is of course rampant. As with other life history traits, it is logical to conclude that resistance is traded off against investment in other important characters, such as development time [7]–[9]. Evolution has not perfected the ability to fend off parasites—i.e., produce organisms that are completely parasite-free—because resources are better expended on other physiological processes.

Both of these frameworks—sexual selection and life history—have led to a series of models and predictions about which species should exhibit more vulnerable males and under what circumstances exceptions might occur [10]. In an earlier paper [11], I suggested that in those species where male fitness is particularly tied to maximizing mating success (i.e., polygynous species, in which a single male may mate with multiple females), males may benefit from sacrificing defense against disease if those resources can instead be devoted towards mating efforts. In monogamous species, males and females typically maximize fitness by assisting in the rearing of offspring. Males and females from monogamous species should then have similarly effective defenses, but as the mating system departs further from monogamy towards polygyny (meaning that the strength of sexual selection on males increases), the sex differences in immune defenses, with males showing the less effective defenses, should increase [11].

## Will the Real Weaker Sex Please Lie Down?

Testing the predictions of this hypothesis has been difficult, partly because data on mating systems of many animals are unavailable and partly because the results of tests are sometimes equivocal. For example, Poulin [12] found evidence for male-biased parasitism in birds when the prevalence of helminth infections was considered, but not when the intensity of infection was considered. Moore and Wilson [13] examined the relationship between sexual selection and parasitism across mammals. Using methods that controlled for correlations between traits due to shared ancestry, Moore and Wilson [13] used two measures of the strength of sexual selection—mating system and sexual size dimorphism—to determine if sexual selection was associated with sex differences in parasitism. As predicted, increases in polygyny or greater male size were associated with greater sex differences in parasitism.

A recent mathematical model [14] allows survival to play an important role in the fitness of both sexes and acknowledges that parasites have sublethal effects that may differ between the sexes. In addition, the model allows the effects of parasitism to be realized through the effects of general health on the traits that are important to fitness. Stoehr and Kokko [14] began with an arbitrary resource allocation strategy for a population, given certain parameter values for the strength of sexual selection, the impact of parasites on condition, and the condition-dependence of reproductive effort. Then, new resource allocation strategies were explored, and any that resulted in higher fitness could “invade” and replace the old strategy; when the best strategy to adopt is the existing strategy, the evolutionarily stable (i.e., “best”) strategy has been achieved.

**Figure 1:**
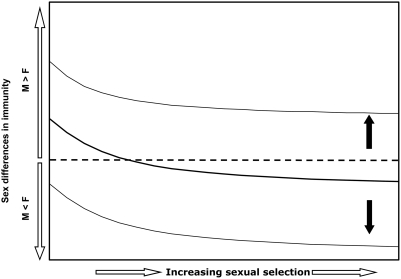
Sex differences in Immunity as a Function of Sexual Selection. The thick solid line represents the case when the condition-dependence of reproduction and the effect of immunity on condition are equal for the sexes; under these circumstances, when sexual selection is absent or weak, males should invest more in immune defense than should females (i.e., thick, solid black line is above the dashed line, in the region of M>F investment in immunity). As the strength of sexual selection increases, the female bias in investment in immunity increases. However, if parasites have particularly strong negative effects on condition in males, and/or if male reproductive success is highly dependent on condition, relative to those same effects in females, males should invest more in immunity than should females, even when sexual selection is strong (thin solid line raised above the thick solid line, and never crossing dashed line). Of course, the converse situation may mean that males never invest more in immunity than do females (lower thin solid line). Adapted from Stoehr and Kokko [14].

Stoehr and Kokko [14] found that, as predicted, the magnitude of the difference between sexes, with males showing an inferior immune response, increases with stronger sexual selection, provided that a) the impact of parasites on condition is the same for the sexes; b) the condition-dependence of reproductive effort is the same for the sexes; and c) neither of these effects is particularly strong. If instead parasites are very detrimental to condition and/or reproductive effort is highly dependent on condition, then males cannot afford to sacrifice immune defense to improve mating success, even in the face of very strong sexual selection. As a result, both sexes invest in immune defense equally. More importantly, the model shows that if the impact of parasites on condition is greater for males than for females, males should invest more of their resources into immune defense than should females, despite intense selection pressure on males to compete for mates (Figure 1). In other words, even if sexual selection causes male investment in immunity to fall below that which would occur in the absence of sexual selection altogether, this decline may still not be sufficient to cause males to invest less in immunity than do females (Figure 1, upper thin solid line).

These findings, both empirical and theoretical, support the idea that sex differences in disease can be most profitably understood in an evolutionary framework. The challenge now is to understand exactly how the differences matter. When we understand the theory and view the mechanisms in that context, we will be able to see why sex differences in disease susceptibility are sometimes male-biased and at other times female-biased. A seldom-considered effect of females possessing a more robust immunity is their increased vulnerability to autoimmune disorders. Whether this represents evolution “over-shooting” to produce a too-vigilant surveillance system is an intriguing but as yet untested possibility. Further work on the evolution of the immune system itself may elucidate this issue [15].

## A Permanent Gender Gap

The discovery that males from many species evolved to be sicker, or at least more susceptible, leads to several considerations, some practical and some theoretical. The first practical consideration is simple: if men and women differ in their response to infection, or in their exposure to it in the first place, it makes sense to tailor treatments accordingly, so that all subpopulations are surveyed appropriately, for example. This recognition is starting to be implemented in the diagnosis and treatment of some health risks such as heart disease, where women are known to experience different symptoms than men, but it is surprisingly absent in consideration of other diseases, particularly infectious ones. Drug treatments should always be tested in both sexes; this may be particularly important in developing nations where parasitic diseases such as intestinal worms that differ in the sexes are more common and where medical resources are particularly limited.

Both longevity itself and the difference between male and female lifespan and susceptibility to parasites are part of who we are. The World Bank projected a closing of the gender gap in longevity by 2025, at least in developed nations, but I suspect this is wildly optimistic at the very least, and more likely utterly hopeless. The gap cannot close easily or quickly because it is the product of a complex framework shaped by evolution. This complexity is why no one will ever be able to point to a single cause for women living longer, whether it is smoking, alcohol abuse, heart disease, infectious disease, or homicides. The same process that gave us men with beards has also made those men die earlier than women. Evidence suggests, for example, that early humans were at least moderately polygynous, so a gender gap in parasite burden is expected in humans as well as other mammals. A promising area for future research lies in connecting understanding of human evolutionary history with current patterns of infection.

None of this is to say that we should give up and let males smoke, drink, or infect themselves to death. It is just that there is nothing “unnatural” about a sex difference in longevity, nothing that is due to a newfangled blip on the biological radar. Before smoking was invented, before motorcycles, before cholesterol-laden cheeseburgers, men were probably more likely to be eaten by saber-toothed tigers. This doesn't mean we shouldn't try and extend life, just as we try to cure disease. But we shouldn't have unrealistic notions about what we are likely to accomplish.
